# A Real-Time Web-Based Intervention with a Multicomponent Group-Based Program for Older Adults: Single-Arm Feasibility Study

**DOI:** 10.3390/healthcare12232365

**Published:** 2024-11-26

**Authors:** Tsubasa Nakada, Kayo Kurotani, Takako Kozawa, Satoshi Seino, Shinichi Murota, Miki Eto, Junko Shimasawa, Yumiko Shimizu, Shinobu Tsurugano, Fuminori Katsukawa, Kazunori Sakamoto, Hironori Washizaki, Yo Ishigaki, Maki Sakamoto, Keiki Takadama, Keiji Yanai, Osamu Matsuo, Chiyoko Kameue, Hitomi Suzuki, Kazunori Ohkawara

**Affiliations:** 1 Graduate School of Informatics and Engineering, The University of Electro-Communications, Tokyo 182-8585, Japan; tsubasanakada@uec.ac.jp (T.N.);; 2 Graduate School of Life Sciences, Showa Women’s University, Tokyo 154-8533, Japan; 3 Faculty of Human Health, Komazawa Women’s University, Tokyo 206-8511, Japan; 4 Institute of Well-Being, Yamagata University, Yamagata 990-9585, Japan; 5 Faculty of Humanities and Social Sciences, Tokyo Metropolitan University, Tokyo 192-0397, Japan; shin1@tmu.ac.jp; 6 Faculty of Human Sciences, Osaka University of Economics, Osaka 533-8533, Japan; eto@osaka-ue.ac.jp; 7 School of Nursing, The Jikei University, Tokyo 182-8570, Japan; 8 Center for Health Sciences and Counseling, Kyushu University, Fukuoka 819-0395, Japan; tsurugano@chc.kyushu-u.ac.jp; 9 Sports Medicine Research Center, Keio University, Kanagawa 223-8521, Japan; fuminori@keio.jp; 10 Green Computing Systems Research Organization, Waseda University, Tokyo 169-8050, Japan; exkazuu@gmail.com; 11 Faculty of Science and Engineering, School of Fundamental Science and Engineering, Waseda University, Tokyo 169-8050, Japan; washizaki@waseda.jp; 12 Research Center for Realizing Sustainable Societies, The University of Electro-Communications, Tokyo 182-8585, Japan; 13 Information Technology Center, The University of Tokyo, Chiba 277-8582, Japan; 14 Office for Research Management, The University of Electro-Communications, Tokyo 182-8585, Japan; suzuki.hitomi@uec.ac.jp

**Keywords:** frailty, online, multicomponent intervention, physical activity, older adults

## Abstract

**Background/Objective:** Frailty is a growing public health challenge in Japan’s rapidly aging population, where 28.8% are aged ≥ 65. While multicomponent interventions have shown potential in preventing frailty, traditional face-to-face programs face accessibility challenges. This study evaluated the feasibility and short-term changes of an online multicomponent intervention on frailty-related factors among community-dwelling older adults. **Methods:** In this single-arm feasibility study, 132 participants (mean age 75.7; standard deviation 4.8 years, 65.2% women) completed a six-week online intervention combining exercise, nutrition education, cognitive activities, and the Coimagination Method to foster social connections, meeting weekly for 75 min sessions in groups of up to 10 participants. **Results:** The intervention demonstrated feasibility with a 96.4% retention rate and a 94.0% average participation rate. While no significant changes were observed in physical activity levels, autonomic nervous system indicators, or cognitive function, carotenoid scores and hemoglobin concentration improved significantly, with more pronounced improvements among women than men. **Conclusions:** This study demonstrates the high feasibility of online multicomponent interventions for older adults and suggests potential benefits for nutritional status markers, particularly among women. These findings indicate a promising and accessible approach to frailty prevention, though randomized controlled trials with longer intervention periods and direct frailty assessments are required to establish effectiveness conclusively. Study Trial registration: UMIN Clinical Trials Registry (UMIN000053089).

## 1. Introduction

Global population aging represents a considerable demographic transition, with projections indicating that the population aged 65 years and older will reach 2.2 billion by the late 2070s [1]. In this context, frailty has emerged as a critical public health challenge worldwide. Frailty is characterized by increased vulnerability to stressors owing to age-related decline in physiological reserves [2]. This multidimensional syndrome, encompassing physical, psychological, and social components [3], is associated with elevated risks of adverse outcomes, including disability and mortality [2,4]. The prevalence of frailty among community-dwelling older adults exhibits substantial international variation, ranging from 4.9% to 27.3% [5] and reflecting diverse demographic characteristics, assessment methodologies, and healthcare systems across regions. Japan, experiencing one of the world’s most accelerated rates of population aging, presents a particularly compelling case. The population aged ≥ 65 years reached 36.4 million in 2020, constituting 28.8% of the total population, with projections indicating an increase to 38.4% by 2065 [6]. While previous epidemiological data reported a frailty prevalence of 7.4% [7], recent evidence from a Japanese longitudinal study demonstrates that the coronavirus disease 2019 (COVID-19) pandemic was associated with an increased prevalence of frailty among community-dwelling older adults [8]. Given the projected dramatic increase in the absolute number of older adults, the prevention and management of frailty remain paramount public health priorities.

Traditional face-to-face programs have demonstrated efficacy in mitigating frailty risk. Physical activity programs have shown particular potential, with high levels of physical activity significantly associated with reduced frailty risk [9]. Additionally, resistance training has emerged as the most efficacious training modality [10]. Similarly, nutritional education programs have yielded positive outcomes in frailty management [11]. Several randomized controlled trials have investigated multicomponent face-to-face programs incorporating exercise and nutritional elements, specifically targeting pre-frail and frail older adults [12,13,14]. Notable among these, a comprehensive program combining exercise, nutritional education, and psychosocial programming demonstrated significant improvements in frailty status [12]. Furthermore, a study focusing on pre-frail and frail community-dwelling older adults established that combined physical, nutritional, and cognitive components exhibited superior efficacy in frailty reduction compared to single-component approaches [13]. Recent systematic evidence suggests that such multimodal face-to-face programs tend to demonstrate greater effectiveness than single-domain approaches in ameliorating frailty status among pre-frail and frail older adults [15].

However, traditional face-to-face programs present substantial limitations regarding accessibility and professional resource allocation. Many older adults encounter barriers to participation due to mobility constraints [16] or geographic impediments [17]. Additionally, the availability of programs is often constrained by limitations in professional staffing and temporal resources. Online multicomponent programs offer potential solutions to these challenges while facilitating broader dissemination of frailty prevention programs. Recent systematic reviews indicate that synchronous online exercise programs may facilitate improvements in physical function [18]. Nevertheless, existing online approaches for mitigating frailty have been limited to asynchronous methods such as video recordings and mobile applications [19]. Despite the potential benefits of such synchronous programs for older adults, the effectiveness of real-time online multicomponent programs specifically designed for frailty prevention among healthy community-dwelling older adults remains largely unexplored.

This study aimed to evaluate the feasibility of a real-time online multicomponent program and examine its short-term effects on frailty-related factors among community-dwelling older adults. The findings are expected to provide insights into the design and implementation of online frailty prevention programs and serve as a foundation for future large-scale randomized controlled trials.

## 2. Materials and Methods

### 2.1. Study Design

This study was conducted as part of the Chofu-Digital-Choju (CDC; Choju means longevity in Japanese) movement, a community-based research initiative conducted from January 2022 to March 2024. The present study is a single-arm, pre–post comparison pilot study designed to assess the feasibility of the intervention. The trial is registered in the UMIN Clinical Trials Registry (UMIN000053089).

### 2.2. Intervention

The online classes integrate a multicomponent intervention approach that combines exercises, nutrition education, and cognitive activities that effectively prevent frailty [13]. Additionally, the classes entailed the Coimagination Method to foster formal conversation techniques aimed at building relationships among participants [20,21]. The weekly online classes included exercises, nutrition education, cognitive activities, and the Coimagination Method, with participants joining from their homes or community centers using tablet devices provided and set up by the research team. Technical support was provided to ensure that all participants could access and effectively navigate the online platform.

The Coimagination Method was designed to promote equitable participation in communication and was conducted in smaller subgroups of approximately five participants within each main group. Facilitators were assigned to each subgroup to encourage communication. Prior to the class, participants submitted their photographs via a chat application or email. These photographs were subsequently displayed on the screen during the online classes. The method comprised two phases: in the first phase, participants explained their preprepared photos; in the second phase, they answered questions from other participants. The questions and comments were posed by the facilitators themselves, by the facilitators asking other participants to share their opinions and thoughts, or by the participants themselves. This process was repeated until all participants completed both phases. Each session of the online classes focused on themes such as landscapes, favorite foods, passions, health, and memorable experiences.

The exercise component involved workouts designed by professional instructors based on a 10-item muscle training program validated in the Japanese Oniishi Model [22,23]. This model was derived from the 19-item regimen developed by Fiatarone et al. [24] and focused on fundamental movements such as walking and standing. The specific exercises included breathing, upper body and lower limb stretches, balance exercises, and lower limb muscle training, such as squats. Each exercise session included pre- and post-training assessments to evaluate the effectiveness of the training, including measurements of breathing capacity, hip mobility, balance ability, sit-to-stand ability, and stride length. Moreover, cognitive enhancement activities led by trainers (such as finger exercises and memory training) were conducted as part of the cognitive aspect of the intervention. No specific intensity was designated for the training to ensure safety in the online environment.

The nutrition component was supervised by nutrition experts and included a series of six lectures on diet and food intake strategies for frailty prevention [25]. The lectures cover the importance of nutrition in frailty prevention, the significance of consuming a diverse range of foods and how to achieve this, the importance of protein intake and strategies to increase it, and the benefits of communal dining. The lectures revolve around a mnemonic phrase in Japanese, “sa-a-ni-gi-ya-ka-ni-i-ta-daku” (‘Let’s eat with diversity’), which emphasizes the importance and techniques of consuming a variety of food groups to prevent frailty [26].

The intervention was conducted in groups of up to 10 participants per group. Each group met once a week for 75 min in the morning, over a period of 6 weeks. Each 75-min class session was structured with 45 min of exercise (including cognitive activities), 10 min of nutrition education, and 15 min of the Coimagination Method, with short breaks between the components. A total of 23 groups completed the intervention, which was repeated throughout the study period.

### 2.3. Participants

Community-dwelling older adults aged 65–84 years were recruited for this study. Exclusion criteria included the need for long-term care or support [27], as well as the presence of medical conditions that would limit the ability to engage in physical exercise, such as severe cardiovascular disease, uncontrolled hypertension, or recent musculoskeletal surgery. Participants were screened using an established questionnaire [28], and those who met any of the exclusion criteria were asked to consult with their primary care physician to determine their eligibility for the study. Participants were recruited through postal mail, local government newsletters, and outreach via social welfare councils and local community associations. As participation in the study was voluntary, our sample likely represented older adults who were interested in and motivated to participate in health promotion activities. The primary objective of this study was to ascertain the feasibility of this intervention. Consequently, no sample size calculations were performed.

### 2.4. Data Collection

Outcome measures were collected in person before and after the intervention at local community centers. The initial assessment involved determining participants’ eligibility and collecting sociodemographic data. After providing a thorough explanation of the study, informed consent was obtained from eligible participants who voluntarily agreed to participate in the research. Subsequently, the outcome measures described below were assessed. Only outcome measurements were conducted during the final assessment.

### 2.5. Outcomes

The study examined two distinct aspects: program feasibility and participant outcomes. Program feasibility was evaluated through retention and participation rates, which serve as key indicators for assessing the potential implementation of larger-scale trials [29]. Participant outcomes were assessed through frailty-related measures including physical activity [10,30], carotenoid levels [31], hemoglobin concentration [32], autonomic nervous system balance [33], and cognitive function [34]. These outcomes were selected based on their established associations with frailty prevention and progression in previous research [10,30,31,32,33,34].

#### 2.5.1. Feasibility Measures

Study feasibility was evaluated by participant retention and intervention adherence. The retention rate was defined as the percentage of participants who completed both the intervention and the subsequent follow-up assessment. Intervention adherence was quantified by calculating the mean attendance rate for the weekly online sessions among those participants who completed the follow-up assessment.

#### 2.5.2. Physical Activity

Physical activity was assessed using a triaxial accelerometer (Active style pro HJA-750c; Omron Healthcare, Kyoto, Japan) with validated measurement accuracy [35,36]. Participants were asked to wear the accelerometer during the intervention period, with the first two weeks from the start of the intervention serving as the baseline period and the last two weeks of the intervention period serving as the follow-up period. However, participants were instructed to remove the device during sleep and bathing. A valid day has at least 10 h of wear time [37,38], and valid data were obtained from participants who had at least one valid day each for weekdays and weekends/holidays. Physical activity was aggregated into light physical activity (LPA; 1.6–2.9 METs), moderate physical activity (MPA; 3.0–5.9 METs), vigorous physical activity (VPA; ≥6.0 METs), and moderate-to-vigorous physical activity (MVPA; ≥3.0 METs). Additionally, daily step counts were assessed.

#### 2.5.3. Skin Carotenoid Level

Skin Carotenoid Level was assessed using a Veggie Meter (Longevity Link Corporation, Salt Lake City, UT, USA), a simple and non-invasive tool that measures the carotenoid levels in the skin using pressure-mediated reflection spectroscopy [39]. Carotenoid levels in the skin correlate with vegetable and fruit intake [40,41]. Measurements were taken from the palm of the hand using the Veggie Meter, and the average of three measurements was used as the outcome indicator.

#### 2.5.4. Hemoglobin Concentration

Hemoglobin concentration was measured non-invasively using the Astrim Fit device (Sysmex, Kobe, Japan). This device optically captures venous blood vessels and measures the hemoglobin concentration per unit volume of blood [42,43].

#### 2.5.5. Autonomic Nervous System Balance

Autonomic nervous system balance was assessed by measuring pulse waves using a TAS9VIEW^®^ pulse oximeter (YKC, Tokyo, Japan). The measurement time was 2 min and 30 s. This device analyzes the volume changes of arterioles at the fingertips as waveforms, enabling the display of numerous indices based on the average waveform type and the fluctuation range of pulse intervals [44]. Autonomic nervous system activity was analyzed based on heart rate variability (HRV) using time-domain and frequency-domain analyses. The root mean square of successive differences (RMSSD) was adopted in the time-domain analysis. RMSSD is associated with parasympathetic nervous activity. A decrease in RMSSD was interpreted as reduced parasympathetic nervous activity [45].

#### 2.5.6. Cognitive Function

Cognitive function was assessed using the “nou-Know” (Eizai, Tokyo, Japan) test, which is based on the Cogstate Brief Battery (Cogstate Ltd., Melbourne, Australia) [46,47]. The “nou-Know” test is a computerized cognitive assessment tool developed for the Japanese population, consisting of four tasks: the Detection Task (DET), Identification Task (IDN), One Card Learning Task (OCL), and One Back Task (ONB). These tasks evaluate psychomotor function, attention, visual learning, and working memory, respectively. The concentration and memory scores measured using “nou-Know” were used as outcome indicators.

### 2.6. Statistical Analysis

Participant characteristics at baseline were described using means and standard deviations (SD) for continuous variables and frequencies with percentages for categorical variables. To compare characteristics between men and women, the Mann–Whitney U test was used for continuous variables and the Chi-square test for categorical variables.

Outcomes were presented as means and SD at baseline and follow-up, along with the mean differences between baseline and follow-up and their 95% confidence intervals (CIs). Physical activity was calculated as a weighted average (five weekdays and two holidays) per day based on valid days for each of the baseline and follow-up periods. RMSSD was evaluated using the natural logarithm transformation (Ln RMSSD) to normalize the distribution of the data.

To examine the effects of the intervention, paired *t*-tests were conducted for each outcome measure, comparing baseline and post-intervention values. These analyses were performed for the entire sample and separately for men and women to investigate potential sex-specific effects of the intervention. A two-sided *p*-value of below 0.05 was considered statistically significant. The magnitude of the effect sizes was interpreted based on Cohen’s guidelines [48], which classify effect sizes of 0.2, 0.5, and 0.8 as small, medium, and large effects, respectively. All statistical analyses were performed using SPSS Statistics version 29.0 (IBM Corp, Armonk, NY, USA).

### 2.7. Ethical Considerations

This study was approved by the Ethical Review Board of The University of Electro-Communications (approval number: 22040(2)). All participants provided written informed consent prior to enrollment, and their personal information was protected throughout the study. Participants were informed of their right to withdraw from the study at any time without consequence.

## 3. Results

**Figure 1:**
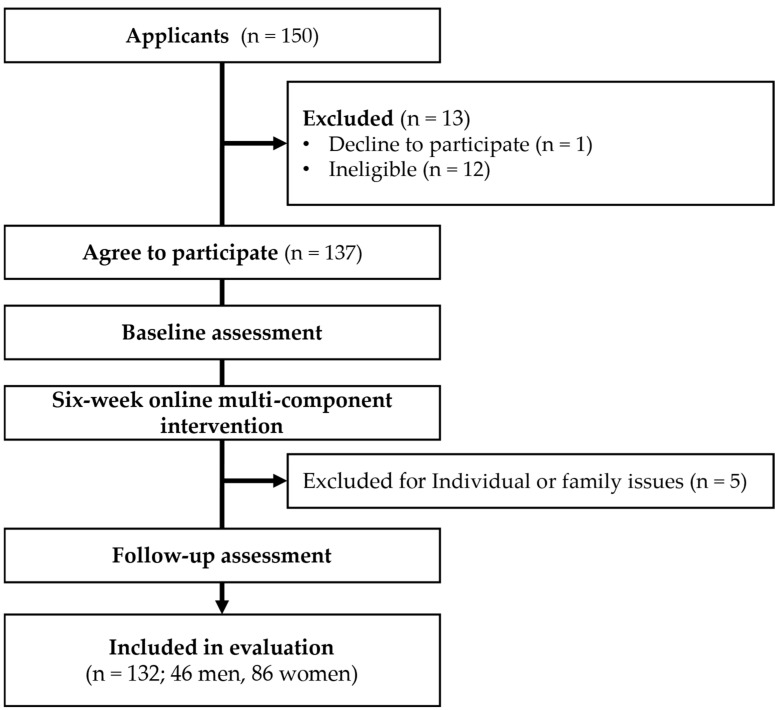
Participant flowchart.

A total of 150 individuals applied for participation through postcards or telephone calls (Figure 1). Among them, 137 individuals (48 men and 89 women) agreed to participate in the study and provided written informed consent. Following this, a baseline survey was conducted, and a follow-up survey was performed after the 6-week program. A total of 132 participants (46 men and 86 women, 65.2% women of all participants) were included in the analysis, excluding five participants who dropped out during the study due to individual or family issues. The retention rate was calculated as 96.4% (132/137). Among the participants who completed the study, the intervention adherence rate ranged from 33% to 100%, with a mean of 94.0%. When stratified by sex, men exhibited an adherence rate ranging from 33% to 100%, with a mean of 92%, while women demonstrated a range of 67% to 100%, with a mean of 95%.

Table 1 presents the demographic and anthropometric characteristics of the participants, stratified by sex. The mean age was 75.7 years (SD: 4.8), with no significant difference between men (75.2 years, SD: 4.9) and women (76.0 years, SD: 4.7; *p* = 0.289). Participants’ mean height was 156.8 cm (SD: 8.9), with men being significantly taller (166.1 cm, SD: 5.9) than women (151.9 cm, SD: 5.7; *p* < 0.001). The average weight was 57.3 kg (SD: 11.1), with men weighing significantly more (66.5 kg, SD: 9.1) compared to women (52.4 kg, SD: 8.8; *p* < 0.001). The mean body mass index (BMI) was 23.2 kg/m^2^ (SD: 3.5), with men having a significantly higher mean BMI (24.1 kg/m^2^, SD: 2.9) than women (22.7 kg/m^2^, SD: 3.7; *p* = 0.012). BMI distribution analysis revealed that 68.7% (n = 90) of participants were in the normal weight range (18.5–25.0 kg/m^2^), while 23.7% (n = 31) were overweight or obese (≥25.0 kg/m^2^), and 7.6% (n = 10) were underweight (<18.5 kg/m^2^). Although the distribution of BMI categories differed between men and women, this difference was not statistically significant (*p* = 0.182).

Table 2 presents the baseline and follow-up data for all participants, while Table 3 and Table 4 show the data for men and women, respectively. Physical activity levels were assessed using a triaxial accelerometer, with data available for 93 participants. No significant changes were observed in LPA, MVPA, MPA, VPA, or daily step count between baseline and follow-up for the entire sample, men, or women (all *p* > 0.05). The effect sizes for these variables ranged from −0.17 to 0.14 across all groups. Carotenoid scores were measured in all 132 participants. A significant increase in carotenoid scores was observed from baseline to follow-up in the entire sample (mean change = 20.5, 95% CI: 6.5 to 34.6, *p* < 0.01, Cohen’s d = 0.25) and in women (mean change = 27.1, 95% CI: 8.4 to 45.9, *p* = 0.01, Cohen’s d = 0.31), but not in men (*p* = 0.42). Hemoglobin levels were measured in 124 participants. There was a significant increase in hemoglobin levels from baseline to follow-up in the entire sample (mean change = 0.4, 95% CI: 0.1 to 0.6, *p* < 0.01, Cohen’s d = 0.27) and in women (mean change = 0.3, 95% CI: 0.1 to 0.6, *p* = 0.02, Cohen’s d = 0.26). The change in men was marginally significant (mean change = 0.5, 95% CI: 0.0 to 1.0, *p* = 0.06, Cohen’s d = 0.29). Autonomic nervous system function was evaluated using Ln RMSSD in 123 participants. No significant changes were observed in Ln RMSSD for the entire sample (mean change = −0.18, 95% CI: −0.36 to 0.01, *p* = 0.06, Cohen’s d = −0.17), men (mean change = −0.25, 95% CI: −0.60 to 0.09, *p* = 0.14, Cohen’s d = −0.22), or women (mean change = −0.14, 95% CI: −0.35 to 0.08, *p* = 0.22, Cohen’s d = −0.14). Cognitive function was assessed using focus and memory scores. Data were available for 129 and 127 participants for focus and memory scores, respectively. No significant changes were observed in either focus or memory scores between baseline and follow-up for the entire sample, men, or women (all *p* > 0.05). The effect sizes for these variables ranged from 0.00 to 0.12 across all groups.

## 4. Discussion

This study evaluated the feasibility of a synchronous, online, multicomponent intervention and examined its short-term effects on frailty-related factors among community-dwelling older adults. Our findings demonstrate the feasibility of the program, with significant improvements observed in carotenoid levels and hemoglobin concentration, particularly among women. However, no statistically significant changes were observed in physical activity, autonomic nervous system function, or cognitive function.

The high participant retention (96.4%) and average participation (94.0%) rates suggest the feasibility of the program without any financial incentives. These rates compare favorably with dropout and attendance rates reported in previous face-to-face exercise programs (65–85% and 58–77%, respectively) [49] and are consistent with findings from other synchronous online exercise programs [50,51]. These favorable rates may be attributed to efforts taken to minimize technical difficulties, similar to the previous studies. This suggests that structured online delivery may be an effective method for engaging older adults in health-promoting activities, regardless of sex. Notably, in our study population, 46.7% of respondents reported daily internet use [52], a rate comparable to the national average (41.5%; among those aged 75–79 years) for older adults in Japan [53]. This context is crucial for interpreting our results, as it suggests that our findings may be generalizable to the broader Japanese older adult population. The high adherence rates achieved despite moderate levels of internet use indicate that well-designed online programs can effectively engage older adults, regardless of their prior internet familiarity. This is particularly significant in the context of the COVID-19 pandemic, where online programs offer a means to maintain health-promoting activities while adhering to social distancing measures [54].

The significant improvement in carotenoid levels, particularly pronounced among women participants, is another noteworthy finding. This improvement likely results from the synergistic effect of multiple components in our intervention program. Primarily, the nutritional education component appears to have contributed to the enhancement of participants’ dietary habits. The lectures on dietary strategies for frailty prevention emphasized the importance of consuming a diverse range of foods, including fruits and vegetables, as well as the necessity of adequate protein intake. These nutrients have been shown to be particularly crucial in frailty prevention in previous studies [31]. Considering that carotenoids serve as biomarkers for fruit and vegetable consumption [40,41], this educational approach seems to have directly impacted dietary behaviors. Moreover, the Coimagination Method used in this study may have contributed to the improvement in carotenoid levels more than anticipated. During the sessions, participants shared and discussed their personal photographs. Notably, some participants chose to share images of meals or culinary creations. This dialogue about dietary habits, facilitated through visual stimuli, may have reinforced the knowledge gained from nutritional education and provided an opportunity for peer learning regarding healthy eating practices. The pronounced improvement in carotenoid levels among women may be attributed to several factors. Previous research has indicated that women generally demonstrate a greater interest in healthy dietary practices compared to men [55,56]. Furthermore, considering that women often bear primary responsibility for meal preparation in many households [57], they may have had more direct opportunities to implement the knowledge gained from nutritional education. While these findings are promising, limitations in our study design preclude direct identification of causal factors for sex disparities and comprehensive evaluation of overall dietary changes, owing to the absence of direct dietary intake assessments.

In this study, an increase in estimated hemoglobin values was observed following a six-week program. The etiology of anemia in the elderly is primarily categorized into three groups: nutrient-deficiency anemia, anemia of chronic disease, and unexplained anemia, with nutrient-deficiency anemia accounting for approximately one-third of cases in older adults. Nutrient-deficiency anemia is associated with deficiencies in iron, vitamin B12, and folate [58,59]. The observed improvement in hemoglobin concentration suggests that our multifaceted intervention approach, which included nutritional education, may have contributed to the amelioration of nutrient-deficiency anemia. Specifically, the nutritional education component may have enhanced participants’ dietary balance, potentially increasing their intake of iron, vitamin B12, and folate. Previous research has reported associations between decreased hemoglobin levels and declines in physical and cognitive function, as well as frailty [32,60,61]. Therefore, the improvement in hemoglobin concentration observed in this study may have implications for frailty prevention. Notably, female participants exhibited pronounced improvements, which may be attributed to differences in baseline characteristics or sex-specific responses to the intervention. This observation warrants further investigation in future studies. Furthermore, factors such as smoking status and its potential impact on early menopause onset [62], which can influence hemoglobin concentrations, should be considered in subsequent analyses. Notably, the interpretation of these results requires caution, as detailed assessments of participants’ nutritional status and inflammatory markers were not conducted.

Despite the multifaceted nature of our program, which incorporated elements of exercise, nutrition education, and social interaction, no statistically significant changes were observed in physical activity levels, autonomic nervous system function, or cognitive performance. The lack of significant changes in physical activity levels may be attributed to several factors. Primarily, the short intervention period of six weeks might have been insufficient to induce measurable changes in these parameters. While physical activity is known to be effective in preventing and improving frailty [10,30], behavior change may require extended periods to become habitual [63]. Additionally, the baseline step count of our participants, being close to the national average of 5006 steps (SD 3635) [64], may have limited the potential for significant increases in their activity levels. Regarding autonomic nervous system function, as measured by heart rate variability (HRV), the absence of significant changes could be attributed to several factors. Previous research suggests that at least three exercise sessions weekly may be necessary to induce clinically meaningful changes in autonomic function [65]. Our program may not have met this threshold of exercise frequency. Moreover, there have been cases where parasympathetic nervous function indicators, including Ln RMSSD, decreased even after a multicomponent exercise program [66]. This highlights the complex nature of autonomic nervous system responses to interventions, especially in older adults, where age-related decline is a factor. The lack of cognitive improvements observed in our study aligns with previous research indicating that multicomponent interventions effective in improving global cognition in older adults typically involve interventions lasting 12 weeks or longer [67]. Our six-week program period may have been insufficient to capture these subtle changes. Additionally, the effects of multicomponent programs on cognitive function in pre-frail or frail older adults remain unclear [15], which may have contributed to our null findings.

Crucially, to the best of our knowledge, this is the first study to investigate a synchronous online program for frailty prevention, demonstrating its feasibility. The positive impact on carotenoid level and hemoglobin concentration partially supports the notion that group-based multicomponent programs, including physical activity and nutritional components, can be effective in preventing frailty, even when delivered online. This approach could be particularly valuable for reaching older adults living in rural areas or those with mobility limitations. However, several limitations of the study must be acknowledged. As a single-arm study without a control group, definitively attributing the observed changes to the intervention itself is challenging. The baseline measurements themselves may have influenced participants’ behaviors and subsequent outcomes. Furthermore, the online format may have attracted participants who are relatively healthy and have higher socioeconomic status [68,69,70], limiting the generalizability of our findings. A significant limitation is the lack of measurement of crucial sociodemographic indicators, such as participants’ socioeconomic status and detailed medical history. The inclusion of these data would have allowed for a more accurate characterization of the participant population and facilitated a more nuanced interpretation of our results. Furthermore, the use of non-invasive methods for measurements, particularly for carotenoid levels and hemoglobin concentration, imposes certain constraints on the accuracy of our data. These measurement methods may have larger margins of error compared to traditional invasive methods, necessitating cautious interpretation of the results. Another critical limitation of this study is that despite the intervention being designed as a multicomponent program for frailty prevention, we did not directly measure the frailty status of participants. The absence of a specific frailty assessment tool limits our ability to draw conclusions about the program’s direct impact on frailty prevention or improvement. Future studies should incorporate validated frailty measures to more accurately assess the effectiveness of the program on frailty outcomes. The 6-week intervention period may have been insufficient to detect changes in certain outcomes, such as physical activity and cognitive function. Notably, this study was conducted as a feasibility trial without adjusting the significance levels across multiple outcomes, so some results may have occurred by chance.

Future research should address these limitations by conducting randomized controlled trials with longer intervention periods and including frail and pre-frail older adults. The use of precise measurement techniques and the inclusion of validated frailty assessment tools should be considered to enhance the reliability of key biomarkers and directly measure frailty outcomes. Further exploration is warranted to investigate the effects of online programs on various populations and to optimize intervention strategies for maximum effectiveness. Additionally, future studies should consider incorporating frequent and intensive exercise sessions, and long follow-up periods to capture potential changes in physical activity, autonomic nervous system function, cognitive performance, and frailty status.

## 5. Conclusions

This study demonstrates the high feasibility of real-time web-based multicomponent interventions as a novel approach to frailty prevention, evidenced by exceptional retention and participation rates. While improvements in physical activity and cognitive function were not observed during the 6-week program, the significant positive changes in hemoglobin concentration and carotenoid levels, particularly among female participants, suggest potential benefits of such programs. These findings are particularly significant given the growing need for accessible frailty prevention strategies among the aging population. Online programs offer a promising and accessible avenue for promoting healthy aging, though future research should employ randomized controlled designs with longer intervention periods and direct frailty assessments to establish their effectiveness conclusively.

## Figures and Tables

**Table 1:** Baseline characteristics of the participants.

Variables	All (n = 132)		Men (n = 46, 34.8%)		Women (n = 86, 65.2%)	
Age, year, mean (SD)	75.7	(4.8)	75.2	(4.9)	76.0	(4.7)
Height, cm, mean (SD)	156.8	(8.9)	166.1	(5.9)	151.9	(5.7)
Weight, kg, mean (SD)	57.3	(11.1)	66.5	(9.1)	52.4	(8.8)
BMI, kg/m^2^, mean (SD)	23.2	(3.5)	24.1	(2.9)	22.7	(3.7)
<18.5, n (%)	10	(7.6)	1	(2.2)	9	(10.6)
18.5–24.9, n (%)	90	(68.7)	32	(69.6)	58	(68.2)
≥25.0, n (%)	31	(23.7)	13	(28.3)	18	(21.2)

Data are presented as the mean (standard deviation) for continuous variables or number (%) for categorical variables. BMI, body mass index; SD, standard deviation. Missing values were removed. One woman’s weight and BMI measurements were not obtained.

**Table 2:** Outcome measures at baseline and follow-up.

Variables	Baseline		Follow-Up		Change	(95% CI)	Effect Size (Cohen d)	*p*-Value
	Mean	SD	Mean	SD				
Physical activity, n = 93								
LPA, min/day	395.9	150.6	397.9	149.2	2.0	−9.3 to 13.3	0.04	0.724
MVPA, min/day	44.6	29.3	43.2	30.0	−1.3	−4.2 to 1.5	−0.10	0.344
MPA, min/day	44.0	28.8	42.4	29.1	−1.5	−4.3 to 1.2	−0.11	0.278
VPA, min/day	0.6	2.7	0.8	3.5	0.2	−0.1 to 0.4	0.14	0.184
Steps, step/day	5543	2973	5361	2957	−181	−460 to 97	−0.13	0.199
Carotenoid score, n = 132	391.7	113.5	412.3	97.6	20.5	6.5 to 34.6	0.25	0.004
Hemoglobin, n = 124	13.8	1.5	14.2	1.4	0.4	0.1 to 0.6	0.27	0.003
Autonomic nervous system (Ln RMSSD), n = 123	3.73	1.05	3.55	0.99	−0.18	−0.36 to 0.01	−0.17	0.057
Cognitive function								
Focus score, n = 129	19.3	4.9	19.3	4.8	0.0	−0.6 to 0.6	0.01	0.922
Memory score, n = 127	23.4	5.7	24.0	5.4	0.6	−0.3 to 1.5	0.11	0.208

LPA, light physical activity; MVPA, moderate-to-vigorous physical activity; MPA, moderate physical activity; VPA, vigorous physical activity; RMSSD, root mean square of successive differences; SD, standard deviation. Missing values were removed and *p*-values were calculated using a paired *t*-test for continuous variables comparing the baseline and follow-up.

**Table 3:** Outcome measures at baseline and follow-up among men.

Variables	Baseline		Follow-Up		Change	(95% CI)	Effect Size (Cohen d)	*p*-Value
	Mean	SD	Mean	SD				
Physical activity, n = 30								
LPA, min/day	385.0	192.1	386.5	181.7	1.5	−18.9 to 21.9	0.03	0.882
MVPA, min/day	46.8	35.8	46.2	35.9	−0.6	−5.8 to 4.6	−0.04	0.813
MPA, min/day	45.5	34.8	44.6	34.1	−0.9	−5.9 to 4.2	−0.06	0.726
VPA, min/day	1.3	4.6	1.6	5.9	0.3	−0.3 to 0.8	0.17	0.357
Steps, step/day	5937	3658	5753	3423	−184	−677 to 309	−0.14	0.452
Carotenoid score, n = 46	390.3	106.7	398.5	100.7	8.2	−12.1 to 28.5	0.12	0.420
Hemoglobin, n = 45	14.7	1.5	15.2	1.0	0.5	0.0 to 1.0	0.29	0.055
Autonomic nervous system (Ln RMSSD), n = 45	4.04	1.19	3.78	0.99	−0.25	−0.60 to 0.09	−0.22	0.144
Cognitive function, n = 46								
Focus score	19.3	5.1	19.4	4.2	0.1	−0.9 to 1.0	0.02	0.873
Memory score	23.2	5.6	23.9	4.9	0.7	−1.0 to 2.4	0.12	0.412

LPA, light physical activity; MVPA, moderate-to-vigorous physical activity; MPA, moderate physical activity; VPA, vigorous physical activity; RMSSD, root mean square of successive differences; SD, standard deviation. Missing values were removed and *p*-values were calculated using a paired *t*-test for continuous variables comparing the baseline and follow-up.

**Table 4:** Outcome measures at baseline and follow-up among women.

Variables	Baseline		Follow-Up		Change	(95% CI)	Effect Size (Cohen d)	*p*-Value
	Mean	SD	Mean	SD				
Physical activity, n = 63								
LPA, min/day	401.1	127.7	403.3	132.3	2.3	−11.7 to 16.2	0.04	0.747
MVPA, min/day	43.5	26.0	41.8	27.0	−1.7	−5.1 to 1.7	−0.12	0.326
MPA, min/day	43.2	25.8	41.4	26.7	−1.8	−5.2 to 1.6	−0.14	0.287
VPA, min/day	0.2	0.6	0.4	1.3	0.1	−0.1 to 0.4	0.12	0.346
Steps, step/day	5355	2598	5175	2718	−180.0	−527 to 166	−0.13	0.303
Carotenoid score, n = 86	392.5	117.6	419.6	95.7	27.1	8.4 to 45.9	0.31	0.005
Hemoglobin, n = 79	13.3	1.1	13.6	1.2	0.3	0.1 to 0.6	0.26	0.021
Autonomic nervous system (Ln RMSSD), n = 78	3.55	0.92	3.41	0.97	−0.14	−0.35 to 0.08	−0.14	0.219
Cognitive function								
Focus score, n = 83	19.3	4.9	19.3	5.1	0.0	−0.8 to 0.8	0.00	0.990
Memory score, n = 81	23.5	5.7	24.0	5.6	0.5	−0.6 to 1.7	0.11	0.346

LPA, light physical activity; MVPA, moderate-to-vigorous physical activity; MPA, moderate physical activity; VPA, vigorous physical activity; RMSSD, root mean square of successive differences; SD, standard deviation. Missing values were removed and *p*-values were calculated using a paired *t*-test for continuous variables comparing the baseline and follow-up.

## Data Availability

The data presented in this study are available on request from the corresponding author.
